# The triglyceride-glucose index is associated with atherosclerosis in patients with symptomatic coronary artery disease, regardless of diabetes mellitus and hyperlipidaemia

**DOI:** 10.1186/s12933-023-01919-z

**Published:** 2023-08-24

**Authors:** Jiao Li, Zixian Dong, Hao Wu, Yue Liu, Yafang Chen, Si Li, Yufan Zhang, Xin Qi, Liping Wei

**Affiliations:** 1 Department of Cardiology, Tianjin Union Medical Center, Nankai University Affiliated Hospital, Tianjin, 300121 China; 2 Nankai University School of Medicine, Tianjin, 300071 China; 3 School of Graduate Studies, Tianjin University of Traditional Chinese Medicine, Tianjin, 301677 China; 4 Key Laboratory of Bioactive Materials Ministry of Education, College of Life Sciences, and State Key Laboratory of Medicinal Chemical Biology, Nankai University, Tianjin, 300071 China

**Keywords:** Triglyceride-glucose index, Risk factors, Coronary atherosclerosis, Carotid atherosclerosis, Diabetes mellitus, Hyperlipidaemia

## Abstract

**Background:**

Diabetes and hyperlipidaemia are both risk factors for coronary artery disease, and both are associated with a high triglyceride-glucose index (TyG index). The TyG index has been presented as a marker of insulin resistance (IR). Its utility in predicting and detecting cardiovascular disease has been reported. However, few studies have found it to be a helpful marker of atherosclerosis in patients with symptomatic coronary artery disease (CAD). The purpose of this study was to demonstrate that the TyG index can serve as a valuable marker for predicting coronary and carotid atherosclerosis in symptomatic CAD patients, regardless of diabetes mellitus and hyperlipidaemia.

**Methods:**

This study included 1516 patients with symptomatic CAD who underwent both coronary artery angiography and carotid Doppler ultrasound in the Department of Cardiology at Tianjin Union Medical Center from January 2016 to December 2022. The TyG index was determined using the Ln formula. The population was further grouped and analysed according to the presence or absence of diabetes and hyperlipidaemia. The Gensini score and carotid intima-media thickness were calculated or measured, and the patients were divided into four groups according to TyG index quartile to examine the relationship between the TyG index and coronary or carotid artery lesions in symptomatic CAD patients.

**Results:**

In symptomatic CAD patients, the TyG index showed a significant positive correlation with both coronary lesions and carotid plaques. After adjusting for sex, age, smoking, BMI, hypertension, diabetes, and the use of antilipemic and antidiabetic agents, the risk of developing coronary lesions and carotid plaques increased across the baseline TyG index. Compared with the lowest quartile of the TyG index, the highest quartile (quartile 4) was associated with a greater incidence of coronary heart disease [OR = 2.55 (95% CI 1.61, 4.03)] and carotid atherosclerotic plaque [OR = 2.31 (95% CI 1.27, 4.20)] (*P* < 0.05). Furthermore, when compared to the fasting blood glucose (FBG) or triglyceride (TG) level, the TyG index had a greater area under the ROC curve for predicting coronary lesions and carotid plaques. The subgroup analysis demonstrated the TyG index to be an equally effective predictor of coronary and carotid artery disease, regardless of diabetes and hyperlipidaemia.

**Conclusion:**

The TyG index is a useful marker for predicting coronary and carotid atherosclerosis in patients with symptomatic CAD, regardless of diabetes mellitus and hyperlipidaemia. The TyG index is of higher value for the identification of both coronary and carotid atherosclerotic plaques than the FBG or TG level alone.

## Introduction

Coronary artery disease (CAD) is the leading cause of morbidity and mortality worldwide, causing 7.4 million deaths in 2015 [1]. Due to the atherosclerotic process, CAD occurs when the coronary arteries are obstructed and is strongly associated with risk factors. CAD can develop slowly and often without symptoms, which is classified as asymptomatic CAD. The most common symptom is angina, which is defined as chest pain radiating to the shoulders, arms, and jaw [2]. Insulin resistance (IR) has been proven to play an important role in CAD [3]. The triglyceride-glucose (TyG) index, calculated from fasting triglyceride and blood glucose levels, has been suggested to be a more reliable marker than IR for metabolic disorders in recent studies [4, 5].

Recently, the TyG index has been linked to an increased risk of cardiovascular disease in otherwise healthy individuals [6]. da Silva et al. [2]. showed that the TyG index was associated with symptomatic CAD requiring secondary care. Zhao et al. [7] proposed that an elevated TyG index was associated with an increased risk of arterial stiffness and nephric microvascular damage. Some researchers are also studying the effect of the TyG index on the prognosis of patients with cardiovascular diseases [8]. However, the predictive significance of the TyG index in CAD patients has not been determined. Atherosclerosis is the pathological basis of CAD, and coronary artery calcification (CAC) is a risk factor for cardiovascular events [9]. In recent years, research on the TyG index and CAC has also progressed [10, 11]. Therefore, it is necessary to study the relationship between the TyG index and atherosclerosis. Diabetes and hyperlipidaemia are both risk factors for CAD [12, 13], and these two variables are directly related to a high TyG index. In this sense, controlling for these two variables is necessary. Comparing the diagnostic value of fasting blood glucose (FBG) and triglyceride (TG) levels with the TyG index is another important point. Therefore, the main objective of this study was to demonstrate that the TyG index is associated with the degree of atherosclerosis in patients with symptomatic CAD.

## Materials and methods

### Population and study design

From 2016 to 2022, we diagnosed and treated 4829 Chinese patients with symptomatic CAD who were hospitalized at Tianjin Union Medical Center. The presence of angina was the first inclusion criterion for symptomatic CAD, followed by positive findings on previous examinations, such as exercise stress testing, myocardial perfusion imaging, coronary CT angiography, and coronary angiography, or a history of previous interventions, such as angioplasty, stenting or angioplasty [2]. Among these patients, 1785 underwent coronary artery angiography, and 2766 underwent carotid Doppler ultrasound. A total of 1516 of these patients underwent coronary angiography as well as carotid ultrasonography; this population was included in the study. This population was separated into four groups based on TyG index quartile. We also conducted subgroup analyses based on the presence or absence of diabetes and hyperlipidaemia. Hyperlipidaemia was defined by a fasting serum total cholesterol level of more than 5.72 mmol/L or triglyceride level of more than 1.7 mmol/L. Diabetes mellitus was defined by FBG ≥ 7.0 mmol/L, a 2-h serum glucose level ≥ 11.1 mmol/L on oral glucose tolerance testing, or the current use of hypoglycaemic drugs or insulin [14]. Ultimately, the relationship between the TyG index and central or peripheral vascular atherosclerosis in symptomatic CAD patients was examined.

### Data collection

Height and weight were measured, and body mass index (BMI) was calculated as weight divided by height squared. Personal medical histories, including hypertension, diabetes and hyperlipidaemia, were collected by self-reporting. A record of all medications the patient was taking before admission, including antilipemic and antidiabetic agents, was obtained. A smoker was defined as someone who had smoked cigarettes on a regular basis in the previous 6 months. Blood samples were collected from participants who were fasting on the morning of the assessment. Concentrations of FBG, total cholesterol (TC), TG, low-density lipoprotein cholesterol (LDL-C) and high-density lipoprotein cholesterol (HDL-C) were measured using an automatic biochemistry analyser. The TyG index was calculated using the formula Ln (fasting TG (mg/dL) × FBG (mg/dL)/2) [15]. The results of carotid Doppler ultrasound and coronary angiography were determined by trained doctors from the Department of Radiology and Cardiology. The Gensini score was calculated to assess the severity of coronary lesions. The carotid intima-media thickness (IMT) and plaque thickness were measured in patients who underwent carotid Doppler ultrasound.

### Statistical analysis

Continuous variables are expressed as the mean ± standard deviation (SD) or median and interquartile range. For normally and nonnormally distributed data, between-group differences were assessed using an independent-samples t test or the Mann‒Whitney U test, respectively. Categorical variables are presented as numbers (percentages) and were compared by chi-square test. Relationships between the TyG index and different groups of coronary artery angiography and carotid Doppler ultrasound data were examined by multivariate logistic regression. Adjustments were made for variables including sex, age, smoking, BMI, history of hypertension and diabetes, and use of antilipemic and antidiabetic agents. The receiver operating characteristic curve (ROC) and area under the curve (AUC) were used to evaluate the ability of the TyG index to predict atherosclerosis. SPSS statistical software, version 26.0, was used for all statistical analyses (SPSS, Inc., Chicago, IL, USA). *P* values of < 0.05 were considered statistically significant.

## Results

### Baseline characteristics

Data were generated from 1516 patients who underwent coronary angiography and carotid ultrasonography. The median age of the individuals was 64 years, and 48.3% were male. The patients were divided into quartiles based on their TyG index. The demographics and features of the four patient groups are shown in Table 1. The four groups of subjects did not differ significantly in terms of age, sex, platelet count, smoking, or antilipemic agent usage. The highest TyG index quartile had a higher proportion of patients with hypertension, diabetes and antidiabetic agent usage (*P* < 0.05); higher BMI, SBP, DBP, Hb, Hb1Ac, FBG, TG, TC, LDL-C and VLDL-C levels (*P* < 0.05); and lower HDL-C levels (*P* < 0.05).

**Table 1:** Clinical characteristics of individuals by TyG index quartile

	TyG index quartiles				*P*
	T1 (lowest) (n = 379)	T2 (n = 379)	T3 (n = 379)	T4 (highest) (n = 379)	
TyG index	8.3 (8.1, 8.4)	8.7 (8.6, 8.8)^a^	9.1 (9.0, 9.2)^ab^	9.6 (9.4, 9.9)^abc^	0.000
Male (n%)	170 (44.9)	183 (48.3)	190 (50.1)	189 (49.9)	0.443
Age (years)	65 (59, 89)	63 (59, 69)	64 (58, 68)	63 (57, 68)	0.110
BMI (kg/m^2^)	24.0 (22.2, 26.2)	25.4 (23.6, 27.7)^a^	25.5 (23.5, 27.7)^a^	25.7 (23.9, 28.1)^a^	0.000
SBP (mmHg)	130 (120, 140)	130 (120, 140)	135 (124, 143)^a^	138 (124, 150)^ab^	0.000
DBP (mmHg)	75 (70, 80)	76 (70, 82)	78 (70, 84)	80 (70, 87)^abc^	0.001
Hb (g/L)	129 (119, 140)	131 (121, 142)	133 (124, 144)^a^	133 (125, 145)^ab^	0.000
PLT (*10^9^/L)	208 (179, 247)	214 (184, 252)	213 (182, 254)	213 (185, 253)	0.325
Hb1Ac (%)	5.7 (5.5, 5.9)	5.9 (5.6, 6.3)^a^	6.2 (5.8, 6.9)^ab^	6.7 (6.1, 8.0)^abc^	0.000
FBG (mg/dL)	94.3 (88.4, 101.5)	102.6 (94.5, 113.4)^a^	110.5 (97.7, 133)^ab^	136.1 (108.7, 169)^abc^	0.000
TG (mg/dL)	81.5 (69.1, 94.8)	121.4 (109, 133.8)^a^	156.8 (131.1, 179.9)^ab^	232.1 (186.1, 289.7)^abc^	0.000
TC (mmol/L)	4.2 (3.5, 4.8)	4.4 (3.6, 5.1)^a^	4.5 (3.9, 5.3)^ab^	4.7 (4.0, 5.5)^abc^	0.000
LDL‑C (mmol/L)	2.4 (1.9, 3.0)	2.7 (2.1, 3.3)^a^	2.8 (2.2, 3.4)^ab^	2.9 (2.4, 3.5)^ab^	0.000
VLDL-C (mmol/L)	0.4 (0.3, 0.5)	0.6 (0.5, 0.7)^a^	0.8 (0.7, 0.9)^ab^	1.2 (1.0, 1.5)^abc^	0.000
HDL‑C (mmol/L)	1.2 (1.0, 1.4)	1.1 (1.0, 1.3)^a^	1.1 (1.0, 1.2)^a^	1.1 (0.9, 1.2)^ab^	0.000
Smoking (n%)	147 (38.8)	135 (35.6)	149 (39.3)	158 (41.7)	0.379
Hypertension (n%)	193 (50.9)	244 (64.4)	265 (69.9)	285 (75.2)	0.000
Diabetes (n%)	29 (7.7)	77 (20.3)	147 (38.8)	206 (54.4)	0.000
Use of antilipemic agents (n%)	72 (19.0)	88 (23.2)	96 (25.3)	97 (25.6)	0.116
Use of antidiabetic agents (n%)	21 (5.5)	55 (14.5)	124 (32.7)	176 (46.4)	0.000

*T1* the first TyG index quartile, *T4* the fourth TyG index quartile, *BMI* body mass index, *SBP* systolic blood pressure, *DBP* diastolic blood pressure, *Hb* hemoglobin, *PLT* platelet count, *HbA1c* glycosylated hemoglobin A1c, *FBG* fasting blood glucose, *TG* triglyceride, *TC* total cholesterol, *LDL-C* low-density lipoprotein, *VLDL-C* very low-density lipoprotein cholesterol, *HDL-C* high-density lipoprotein

*P* < 0.05 were considered statistically significant

^a^*P* < 0.05 vs. T1

^b^*P* < 0.05 vs. T2

^c^*P* < 0.05 vs. T3

### Relationship between the TyG index and risk factors for cardiovascular disease

**Fig. 1:**
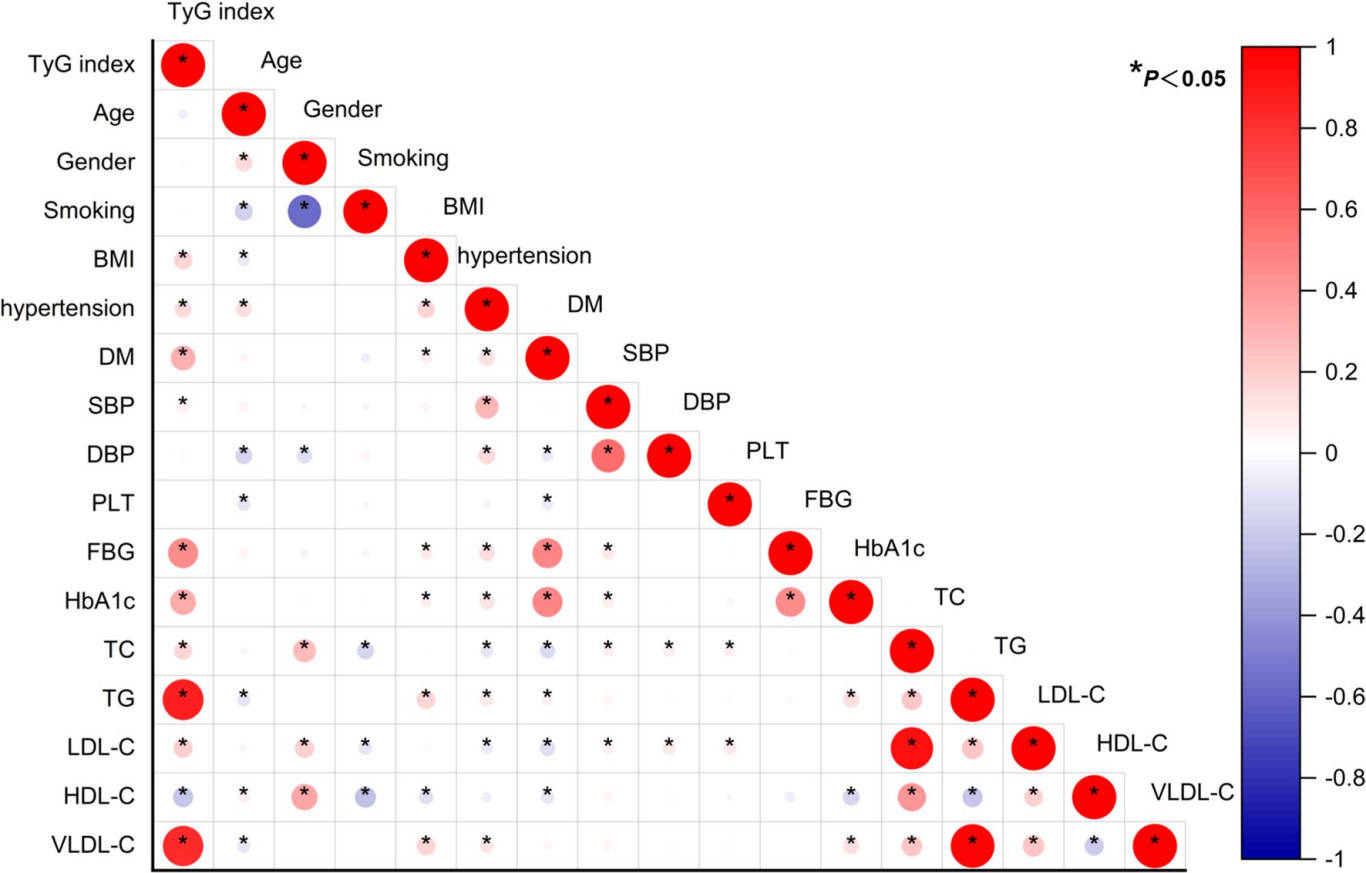
Correction of the TyG index with risk factors for CHD. *P* < 0.05 were considered statistically significant. *CHD* coronary heart disease, *BMI* body mass index, *DM* diabetes mellitus, *SBP* systolic blood pressure, *DBP* diastolic blood pressure, *PLT* platelet count, *HbA1c* glycosylated hemoglobin A1c, *FBG* fasting blood glucose, *TG* triglyceride, *TC* total cholesterol, *LDL-C* low-density lipoprotein, *VLDL-C* very low-density lipoprotein cholesterol, *HDL-C* high-density lipoprotein

Symptomatic CAD patients were separated into two groups based on whether coronary angiography revealed normal coronary arteries or coronary lesions (i.e., coronary heart disease, CHD), as indicated by atherosclerotic stenosis of at least 50% of the diameter of any coronary artery. The results showed a significant difference in sex, age, BMI, TyG index, SBP, TC, and HbA1C between the CHD and normal groups, with higher values in the CHD group, while the HDL-C level was significantly lower in the CHD group (*P* < 0.05) (Table 2). Furthermore, there were significant disparities between the two groups in terms of hypertension, diabetes, smoking, and medication usage (antilipemic and antidiabetic agents) (Table 2). Although there was no significant difference in the LDL-C level, it is known to be a risk factor for CHD and was still included in the following statistical model. We also conducted a correlation study between the TyG index and risk factors for CHD, as shown in Fig. 1. The TyG index correlated positively with BMI, TC, LDL-C, FBG, TG, and a history of hypertension and diabetes but negatively with HDL-C (P < 0.05).

**Table 2:** Clinical characteristics of symptomatic CAD patients based on coronary angiography

	Coronary normal (n = 261)	Coronary lesion (n = 1255)	*P*
Male (n%)	74 (28.4)	658 (52.4)	0.000
Age (years)	61 (56, 66)	64 (59, 69)	0.000
BMI (kg/m^2^)	24.8 (22.9, 27.4)	25.2 (23.4, 27.7)	0.027
TyG index (n%)			0.000
T1 (lowest)	107 (41)	272 (21.7)	
T2	66 (25.3)	313 (24.9)	
T3	51 (19.5)	328 (26.1)	
T4 (highest)	37 (17.2)	342 (27.3)	
SBP (mmHg)	130 (120, 140)	133 (122, 145)	0.000
TC (mmol/L)	4.6 (3.9, 5.2)	4.4 (3.7, 5.2)	0.016
LDL-C (mmol/L)	2.8 (2.2, 3.4)	2.7 (2.1, 3.3)	0.313
HDL-C (mmol/L)	1.2 (1.0, 1.4)	1.1 (1.0, 1.3)	0.000
PLT (*10^9^/L)	209 (182, 248)	213 (183, 252)	0.707
HbA1C (%)	5.8 (5.6, 6.2)	6.1 (5.7, 6.8)	0.000
Hypertension (n%)	134 (51.3)	853 (68)	0.000
Diabetes (n%)	39 (14.9)	420 (33.5)	0.000
Smoking (n%)	72 (27.6)	517 (41.2)	0.000
Use of antilipemic agents (n%)	26 (10)	327 (26.1)	0.000
Use of antidiabetic agents (n%)	28 (10.7)	348 (27.7)	0.000

*T1* the first TyG index quartile, *T4* the fourth TyG index quartile, *BMI* body mass index, *SBP* systolic blood pressure, *TC* total cholesterol, *LDL-C* low-density lipoprotein, *HDL-C* high-density lipoprotein, *PLT* platelet count, *HbA1c* glycosylated hemoglobin A1

*P* < 0.05 were considered statistically significant

### Multivariate logistic regression analysis of CHD by TyG index quartile

Table 3 shows the results of this analysis with normal coronary patients as the reference group. According to the findings, the fourth TyG index quartile was significantly related to a higher incidence of CHD. Additionally, the risk of CHD was 2.55 times higher in the T4 group than in the T1 group, regardless of social characteristics (sex, age), lifestyle (smoking, BMI), and disease features (hypertension, diabetes, use of antilipemic and antidiabetic agents) [OR = 2.55 (95% CI 1.61, 4.03)] (*P* < 0.05).

**Table 3:** Multivariate logistic regression analysis of CHD by TyG index quartile

Coronary angiography	TyG index quartiles			
	T1 (lowest)	T2	T3	T4 (highest)
	OR (95% CI)			
Normal (n = 261) Ref.				
CHD (n = 1255)				
Model 1	Ref.	1.87 (1.32, 2.64)*	2.53 (1.75, 3.66)*	3.64 (2.42, 5.46)*
Model 2	Ref.	1.87 (1.30, 2.69)*	2.50 (1.70, 3.67)*	3.85 (2.53, 5.87)*
Model 3	Ref.	1.64 (1.12, 2.41)*	1.87 (1.24, 2.82)*	2.55 (1.61, 4.03)*

Data are odds ratios (95% CI) of multivariate logistic regression

Model 1: crude

Model 2: sex and age adjustments

Model 3: adjusted for model 2, smoking, BMI, hypertension, diabetes and medication usage (antilipemic and antidiabetic agents)

*CHD* coronary heart disease, *OR* odds ratios, *CI* confidence intervals, *T1* the first TyG index quartile, *T4* the fourth TyG index quartile

^*^P < 0.05

### Association between the TyG index and coronary lesion severity

**Fig. 2:**
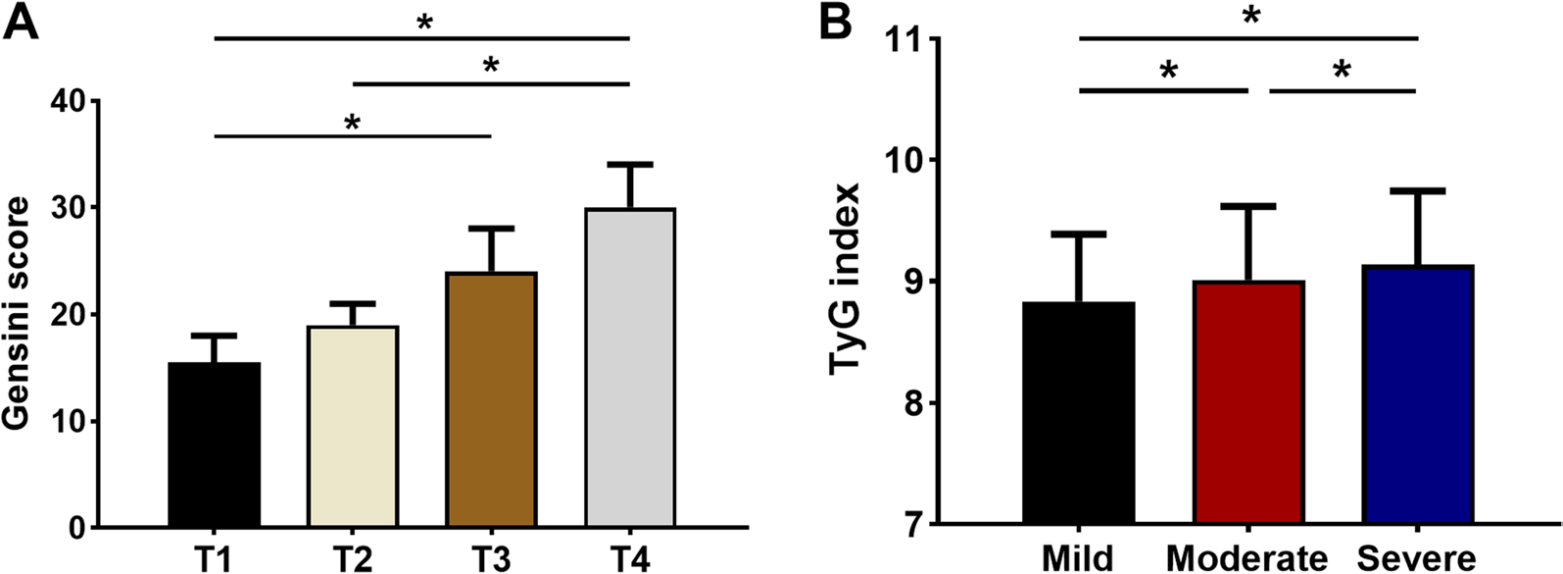
Relationship between Gensini score and the TyG index in CHD patients. **A** The Gensini score by TyG index quartile; **B** the TyG index by Gensini score tertile. *P < 0.05. *T1* the first TyG index quartile, *T4* the fourth TyG index quartile

Patients with CHD were classified based on Gensini scores and coronary lesion branches. These individuals were divided into three groups based on the Gensini score tertile, as follows: mild (tertile 1), moderate (tertile 2), and severe coronary artery lesions (tertile 3). The findings revealed that the severity of coronary artery lesions differed significantly by the TyG index level (Table 4). Figure 2 shows the Gensini score by TyG index quartile as well as the TyG index by Gensini score tertile in CHD patients. Compared with the T1 and T2 groups, the Gensini score was significantly increased in the T4 group (*P* < 0.05). The TyG index was significantly greater in the severe coronary artery lesion group (Gensini score tertile 3) than in the mild and moderate coronary artery lesion groups (*P* < 0.05).

**Table 4:** Association between the TyG index and coronary lesion severity in CHD patients

	TyG index quartiles				*P*
	T1 (lowest) (n = 314)	T2 (n = 314)	T3 (n = 314)	T4 (highest) (n = 314)	
Gensini score (tertiles)					0.000
Mild (n = 418)	136	117	93	72	
Moderate (n = 418)	102	101	109	106	
Severe (n = 419)	76	96	112	135	
Coronary lesion branches					0.000
1-vessel (n = 353)	124	95	79	55	
2-vessel (n = 389)	97	99	90	103	
3-vessel (n = 513)	93	120	145	155	
Left main lesion					0.131
Yes (n = 54)	16	19	8	11	
No (n = 1201)	298	295	306	302	

*CHD* coronary heart disease, *T1* the first TyG index quartile, *T4* the fourth TyG index quartile

*P* < 0.05 were considered statistically significant

### Association between the TyG index and carotid lesions

**Fig. 3:**
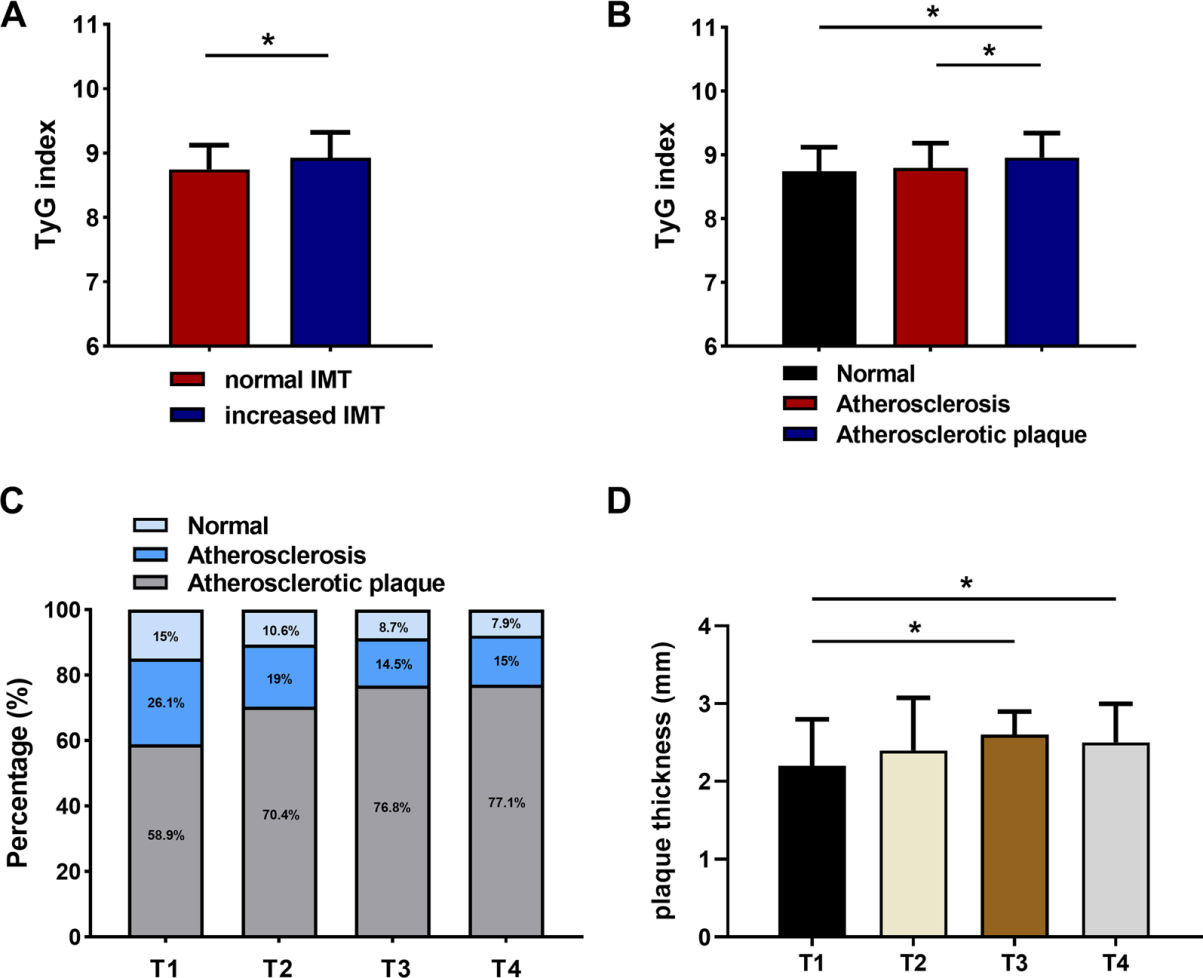
Connection between carotid lesions and the TyG index in symptomatic CAD patients. **A**, **B** The TyG index by carotid lesions groups; **C** proportion of carotid ultrasound findings by TyG index quartile; **D** plaque thickness by TyG index quartile. *P < 0.05. *T1* the first TyG index quartile, *T4* the fourth TyG index quartile

All patients underwent carotid Doppler ultrasound and were classified based on intima-media thickness (IMT), with an IMT greater than 1.0 mm defining carotid artery intima-media thickening. The results revealed that the TyG index was considerably higher in the increased IMT group than in the normal IMT group (*P* < 0.05) (Fig. 3A). Table 5 shows the clinical characteristics of patients undergoing carotid ultrasonography. Patients in the increased IMT group presented significantly higher age, SBP, and HbA1c (%) values and a lower HDL-C value than those in the normal IMT group (*P* < 0.05). There were significant disparities between the two groups in terms of hypertension, diabetes, smoking, and use of antidiabetic agents (*P* < 0.05). However, there was no significant difference in BMI, TC, LDL-C, PLT, or use of antilipemic agents.

**Table 5:** Clinical characteristics of patients grouped by carotid ultrasonography

	Normal IMT (n = 160)	Increased IMT (n = 1356)	*P*
Male (n%)	40 (25.0)	692 (51.0)	0.000
Age (years)	57 (52, 63)	64 (59, 69)	0.000
BMI (kg/m^2^)	24.7 (23.0, 27.3)	25.2 (23.4, 27.7)	0.231
TyG index (n%)			0.007
T1 (n = 552) (lowest)	57 (35.6)	322 (23.7)	
T2 (n = 551)	40 (25.0)	339 (25.0)	
T3 (n = 552)	33 (20.6)	346 (25.5)	
T4 (n = 552) (highest)	30 (18.8)	349 (25.7)	
SBP (mmHg)	130 (120, 140)	133 (122, 144)	0.002
TC (mmol/L)	4.5 (4.0, 5.1)	4.4 (3.7, 5.2)	0.199
LDL-C (mmol/L)	2.7 (2.2, 3.3)	2.7 (2.1, 3.3)	0.967
HDL-C(mmol/L)	1.2 (1.0, 1.4)	1.1 (1.0, 1.3)	0.000
PLT (*10^9^/L)	210 (184, 253)	211 (183, 251)	0.998
HbA1C (%)	5.8 (5.5, 6.2)	6.1 (5.7, 6.8)	0.000
Hypertension (n%)	70 (43.8)	917 (67.6)	0.000
Diabetes (n%)	33 (20.6)	426 (31.4)	0.005
Smoking (n%)	28 (17.5)	561 (41.4)	0.000
Use of antilipemic agents (n%)	28 (17.5)	325 (24.0)	0.067
Use of antidiabetic agents (n%)	26 (16.3)	350 (25.8)	0.008

*T1* the first TyG index quartile, *T4* the fourth TyG index quartile, *IMT* carotid intima-media thickness, *BMI* body mass index, *SBP* systolic blood pressure, *TC* total cholesterol, *LDL-C* low-density lipoprotein, *HDL-C* high-density lipoprotein, *PLT* platelet, *HbA1c* glycosylated hemoglobin A1c

*P* < 0.05 were considered statistically significant

In addition, the increased IMT group was split into two groups, including those with carotid atherosclerosis (IMT greater than 1.0 mm) and carotid plaque formation (IMT not less than 1.5 mm). The TyG index in the carotid plaque group was significantly greater than that in the other group (*P* < 0.05) (Fig. 3B). The largest proportion of patients with carotid plaque was found in the fourth TyG index quartile (Fig. 3C). Assessment of plaque thickness in the carotid atherosclerotic plaque group revealed considerably thicker plaques in the highest TyG quartile than in the lowest quartile (Fig. 3D).

### Multivariate logistic regression analysis of carotid atherosclerotic plaques by TyG index quartile

Table 6 suggests that a significant association between the fourth TyG index quartile and a higher prevalence of carotid plaque. Referencing patients with normal carotid arteries, the risk of carotid plaque in the T4 group was 2.31 times higher than that in the T1 group, regardless of social (sex, age), lifestyle (smoking, BMI) and clinical characteristics features (hypertension, diabetes, use of antilipemic and antidiabetic agents) [OR = 2.31 (95% CI 1.27, 4.20)] (*P* < 0.05).

**Table 6:** Association between the TyG index and carotid atherosclerotic plaques in patients with symptomatic CAD

Carotid Doppler ultrasound	TyG index quartiles			
	T1 (lowest)	T2	T3	T4 (highest)
	OR (95% Cl)			
Normal (n = 160) Ref.				
Atherosclerosis (n = 283)				
Model 1	Ref.	1.04 (0.63, 1.72)	0.96 (0.56, 1.65)	1.09 (0.63, 1.90)
Model 2	Ref.	1.00 (0.59, 1.68)	0.96 (0.55, 1.68)	1.22 (0.69, 2.15)
Model 3	Ref.	0.99 (0.57, 1.74)	0.78 (0.43, 1.42)	0.83 (0.44, 1.56)
Atherosclerotic plaques (n = 1073)				
Model 1	Ref.	1.71 (1.10, 2.66)*	2.25 (1.42, 3.58)*	2.49 (1.55, 4.01)*
Model 2	Ref.	1.77 (1.08, 2.90)*	2.50 (1.49, 4.18)*	3.32 (1.95, 5.67)*
Model 3	Ref.	1.76 (1.03, 3.02)*	2.15 (1.22, 3.77)*	2.31 (1.27, 4.20)*

Data are odds ratios (95% CI) of multivariate logistic regression

Model 1: crude

Model 2: sex and age adjustments

Model 3: adjusted for model 2, smoking, hypertension, diabetes and medication usage (antilipemic agents and antidiabetic agents)

*OR* odds ratios, *CI* confidence intervals, *T1* the first TyG index quartile, *T4* the fourth TyG index quartile

*P < 0.05

### ROC analyses of the TyG index

**Fig. 4:**
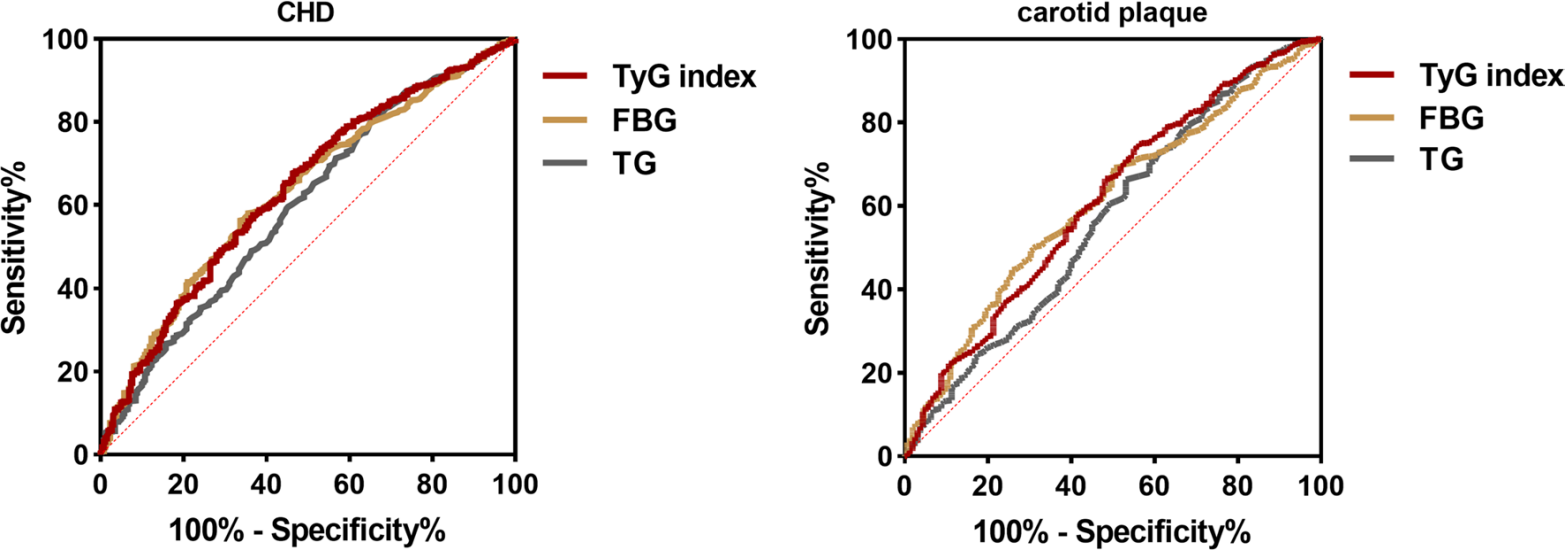
ROC analyses of the TyG index. *CHD* coronary heart disease, *FBG* fasting blood glucose, *TG* triglyceride, *ROC* receiver operating characteristic curve

The results of the ROC analyses of the TyG index, FBG level and TG level are summarized in Table 7. The TyG index showed a larger AUC than the FBG or TG level. The critical TyG index for CHD was 8.727 (sensitivity, 67.6%; specificity, 53.6%), and the critical TyG index for carotid plaque was 8.725 (sensitivity, 69.2%; specificity, 48.3%) (Fig. 4; Table 7).

**Table 7:** ROC analyses of the TyG index

	AUC	95% CI	*P*	Sensitivity	Specificity	Youden’s index
CHD						
TyG index	0.636	0.600–0.673	0.000	0.676	0.536	8.727
FBG (mg/dL)	0.632	0.596–0.668	0.000	0.563	0.663	103.950
TG (mg/dL)	0.598	0.560–0.636	0.000	0.800	0.349	97.017
Carotid atherosclerotic plaque						
TyG index	0.603	0.572–0.635	0.000	0.692	0.483	8.725
FBG (mg/dL)	0.594	0.563–0.625	0.000	0.691	0.476	98.910
TG (mg/dL)	0.574	0.542–0.607	0.000	0.804	0.318	97.903

*CHD* coronary heart disease, *FBG* fasting blood glucose, *TG* triglyceride, *CI* confidence intervals, *ROC* receiver operating characteristic curve, *AUC* area under the curve

*P* < 0.05 were considered statistically significant

### Association between the TyG index and coronary lesions in patients with or without diabetes mellitus

**Fig. 5:**
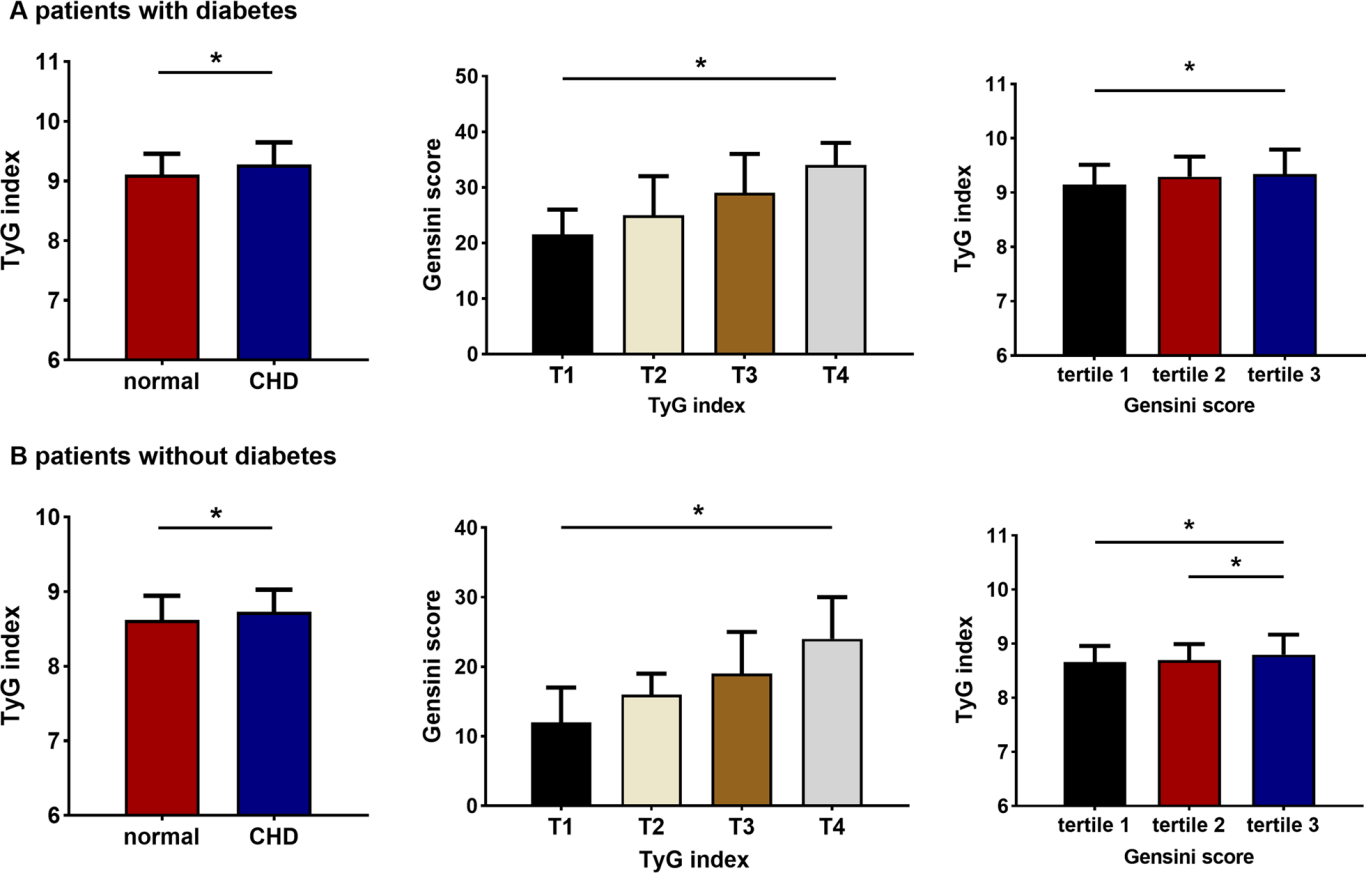
Relationship between the TyG index and coronary lesions in patients with or without diabetes. *P < 0.05. *T1* the first TyG index quartile, *T4* the fourth TyG index quartile, *CHD* coronary heart disease

Patients with symptomatic CAD were divided into subgroups based on the presence or absence of diabetes. Figure 5 demonstrates a relationship between the TyG index and coronary lesions in patients with or without diabetes. It also shows the Gensini score by TyG index quartile as well as the TyG index by Gensini score tertile in CHD patients.

Multivariate logistic regression in diabetic patients revealed a 2.489-fold increase in the likelihood of coronary lesions in the highest TyG index quartile, regardless of sex, age, BMI, smoking, hypertension, and use of antilipemic and antidiabetic agents [OR = 2.489 (95% CI 1.084, 5.716)] (*P* < 0.05) (Table 8). A similar analysis of data from nondiabetic patients with adjustments for confounders showed a 1.932-fold increase in the incidence of coronary lesions in the highest TyG index quartile [OR = 1.932 (95% CI 1.191, 3.133)] (*P* < 0.05) (Table 8).

**Table 8:** OR and 95% CI for the highest versus lowest quartiles predicting the presence of CHD

TyG index quartiles	With DM		Without DM	
	OR (95% CI)	*P*	OR (95% CI)	*P*
Model 1	2.403 (1.093, 5.284)	0.029	1.984 (1.286, 3.062)	0.002
Model 2	2.527 (1.133, 5.638)	0.024	2.106 (1.329, 3.339)	0.002
Model 3	2.489 (1.084, 5.716)	0.032	1.932 (1.191, 3.133)	0.008

Data are odds ratio (95% CI) based in multivariate logistic regression

Model 1: crude

Model 2: sex and age adjustments

Model 3: adjusted for model 2, smoking, BMI, hypertension and use of antilipemic agents and antidiabetic agents

*DM* diabetes mellitus, *OR* odds ratios, *CI* confidence intervals, *CHD* coronary heart disease

*P* < 0.05 were considered statistically significant

### Association between the TyG index and carotid artery lesions in patients with or without diabetes mellitus

**Fig. 6:**
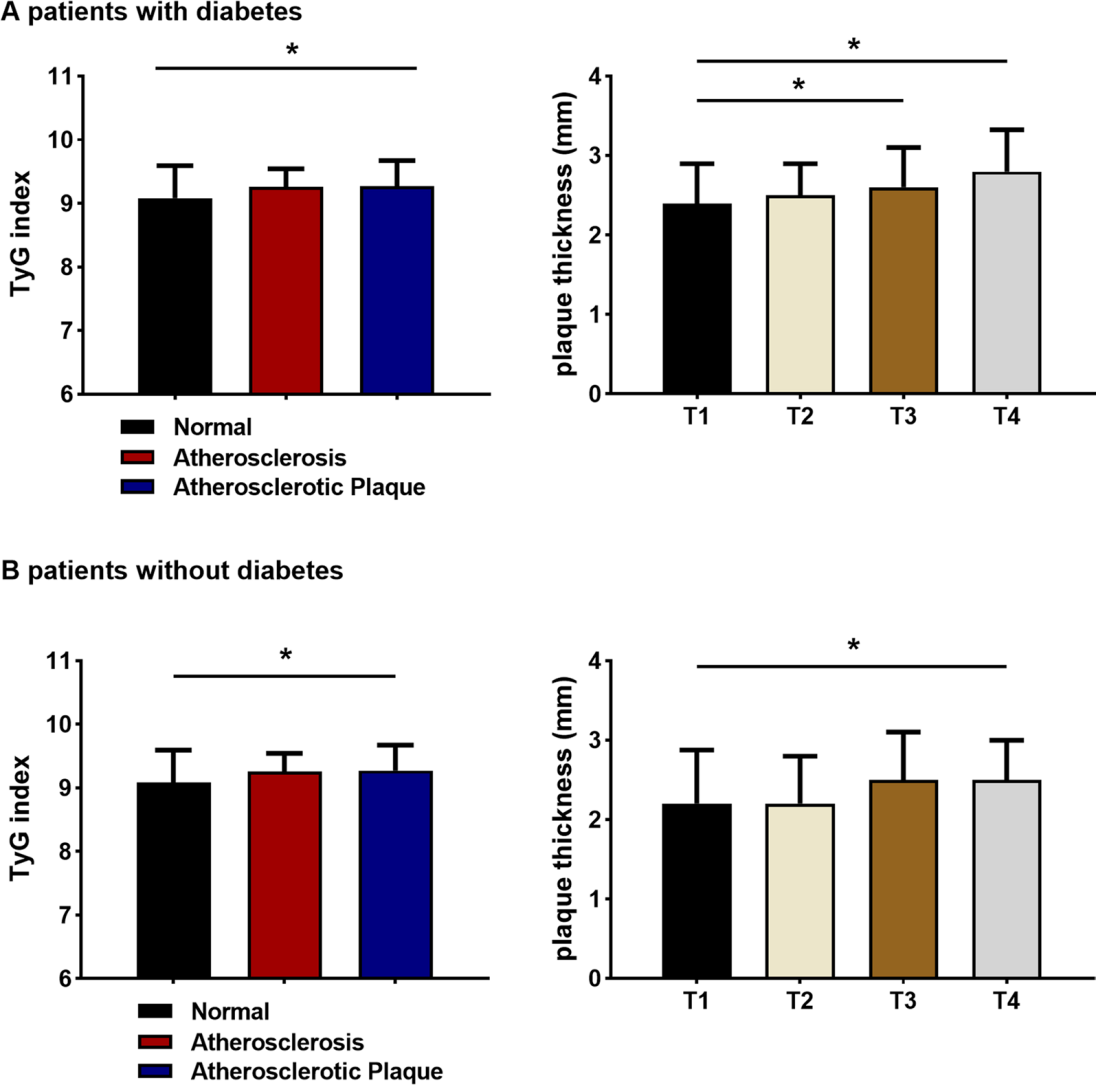
Connection between carotid lesions and the TyG index in patients with or without diabetes. *P < 0.05. *T1* the first TyG index quartile, *T4* the fourth TyG index quartile

Regardless of diabetes, the TyG index and carotid artery lesions were positively associated. Figure 6 reveals that the TyG index was significantly higher in the plaque group than in the normal group (*P* < 0.05). In addition, the plaques in the highest TyG index quartile were substantially thicker than those in the lowest TyG index quartile (*P* < 0.05).

Multivariate logistic regression in diabetic patients who underwent carotid Doppler ultrasonography (Table 9) revealed a 2.872-fold increase in the incidence of carotid atherosclerotic plaque in the highest TyG index quartile, regardless of sex and age (95% CI 1.119, 7.369) (*P* < 0.05). However, after excluding confounding factors (sex, age, smoking, hypertension and use of antilipemic agents), there was no significant difference in carotid plaque formation between the highest and lowest TyG index quartiles [OR = 1.521 (95% CI 0.544, 4.249)]. A similar analysis of data from nondiabetic patients with adjustments for confounders showed a 2.806-fold increase in the incidence of carotid plaque formation in the highest TyG index quartile [OR = 2.806 (95% CI 1.061, 4.098)] (*P* < 0.05) (Table 9).

**Table 9:** OR and 95% CI for the highest versus lowest quartiles predicting the presence of carotid atherosclerotic plaques

TyG index quartiles	With DM		Without DM	
	OR (95% CI)	*P*	OR (95% CI)	*P*
Model 1	1.976 (0.828, 4.714)	0.125	1.621 (0.946, 2.779)	0.079
Model 2	2.872 (1.119, 7.369)	0.028	2.211 (1.180,4.142)	0.013
Model 3	1.521 (0.544, 4.249)	0.424	2.806 (1.061, 4.098)	0.033

Data are odds ratio (95% CI) based in multivariate logistic regression

Model 1: crude

Model 2: sex and age adjustments

Model 3: adjusted for model 2, smoking, BMI, hypertension and use of antilipemic agents

*DM* diabetes mellitus, *OR* odds ratios, *CI* confidence intervals

*P* < 0.05 were considered statistically significant

### Relationship of the TyG index and coronary lesions in patients with or without hyperlipidaemia

**Fig. 7:**
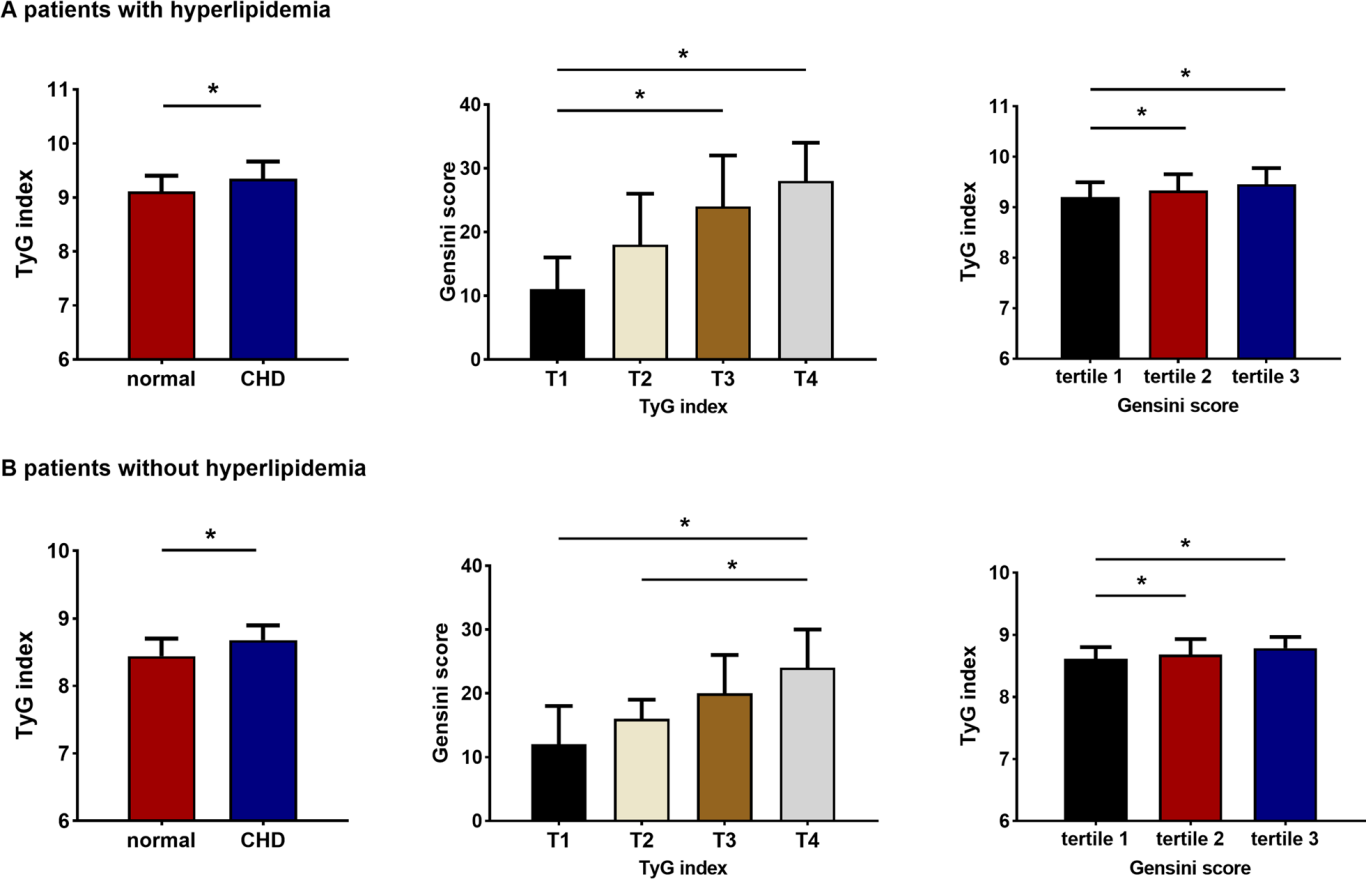
Relationship of the TyG index and coronary lesions in patients with or without hyperlipidaemia. *P < 0.05. *T1* the first TyG index quartile, *T4* the fourth TyG index quartile, *CHD* coronary heart disease

Additional subgroup analyses were conducted depending on the presence or absence of hyperlipidaemia. Figure 7 presents the relation between the TyG index and the severity of coronary lesions in the presence or absence of hyperlipidaemia.

The findings of the multivariate logistic regression analysis in individuals with and without hyperlipidaemia are shown in Table 10. The highest TyG index quartile showed a higher prevalence of coronary lesions. In the hyperlipidaemia group, after adjusting for age and sex, multivariate logistic analysis revealed an OR of 2.982 (95% CI 1.556, 5.717) (*P* < 0.05). Regardless of sex, age, BMI, smoking, hypertension, diabetes, and use of antilipemic and antidiabetic medicines, the OR in patients without hyperlipidaemia was 2.656 (95% CI 1.343, 5.252) (*P* < 0.05).

**Table 10:** Multivariate logistic analysis for the highest versus lowest quartile of the TyG index predicting the occurrence of CHD

TyG index quartiles	With hyperlipidaemia		Without hyperlipidaemia	
	OR (95% CI)	*P*	OR (95% CI)	*P*
Model 1	3.289 (1.741, 6.213)	0.000	4.731 (2.655, 8.432)	0.000
Model 2	2.982 (1.556, 5.717)	0.001	4.086 (2.250, 7.418)	0.000
Model 3	1.611 (0.767, 3.380)	0.208	2.656 (1.343, 5.252)	0.000

Data are odds ratio (95% CI) based in multivariate logistic regression

Model 1: crude

Model 2: sex and age adjustments

Model 3: adjusted for model2, smoking, BMI, hypertension, diabetes and use of antilipemic agents and antidiabetic agents

*OR* odds ratios, *CI* confidence intervals, *CHD* coronary heart disease

*P* < 0.05 were considered statistically significant

### Relationship of the TyG index and carotid artery lesions in patients with or without hyperlipidaemia

**Fig. 8:**
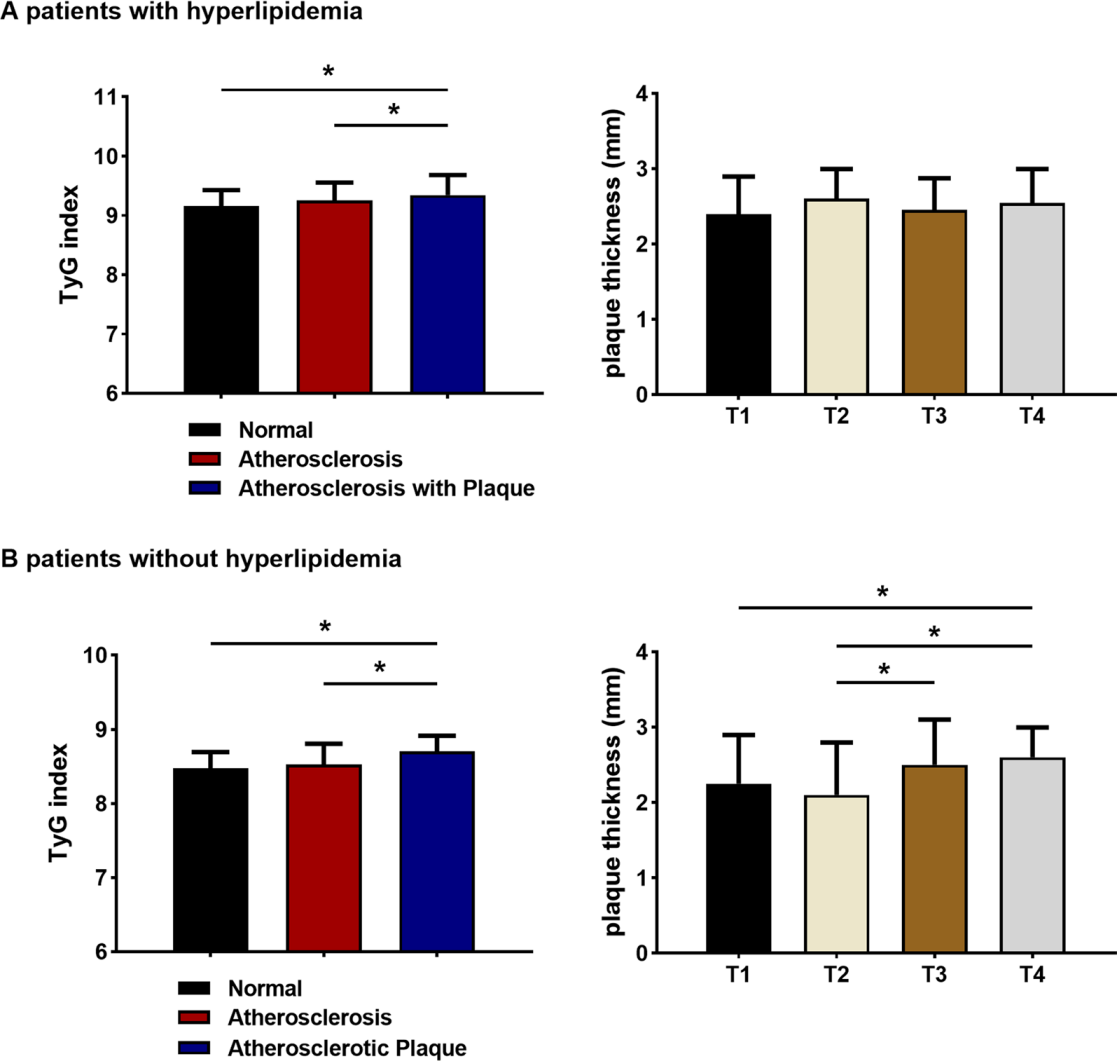
Connection between carotid lesions and the TyG index in patients with or without hyperlipidaemia. *P < 0.05. *T1* the first TyG index quartile, *T4* the fourth TyG index quartile

Regardless of hyperlipidaemia, there was a positive relation between the TyG index and carotid artery lesions. As shown in Fig. 8, the TyG index was significantly higher in the carotid plaque group than in the normal group (*P* < 0.05). The carotid plaque thickness was larger in the T4 group than in the T1 group in those without hyperlipidaemia (*P* < 0.05). However, there was no significant difference in plaque thickness between the groups in the hyperlipidaemic population.

As shown in Table 11, carotid atherosclerotic plaque was 2.969-fold more likely in the highest TyG index quartile regardless of sex and age in patients with hyperlipidaemia (95% CI 1.273, 6.922) (*P* < 0.05). In patients without hyperlipidaemia, the likelihood of carotid plaque formation was 4.715-fold higher in the highest TyG index quartile, with adjustments for sex, age, BMI, smoking, hypertension, diabetes, and use of antilipemic and antidiabetic medicines [OR = 4.715 (95% CI 1.763, 12.612)] (*P* < 0.05) (Table 11).

**Table 11:** Multivariate logistic analysis for the highest versus lowest quartile of the TyG index predicting the occurrence of carotid atherosclerotic plaques

TyG index quartiles	With hyperlipidaemia		Without hyperlipidaemia	
	OR (95% CI)	*P*	OR (95% CI)	*P*
Model 1	2.755 (1.253, 6.058)	0.012	6.296 (2.932, 13.522)	0.000
Model 2	2.969 (1.273, 6.922)	0.012	5.536 (2.397, 12.787)	0.000
Model 3	1.677 (0.628, 4.479)	0.302	4.715 (1.763, 12.612)	0.002

Data are odds ratio (95% CI) based in multivariate logistic regression

Model 1: crude

Model 2: sex and age adjustments

Model 3: adjusted for model 2, smoking, BMI, hypertension, diabetes and use of antilipemic agents and antidiabetic agents

*OR* odds ratios, *CI* confidence intervals

*P* < 0.05 were considered statistically significant

## Discussion

Both insulin resistance (IR) and cardiovascular disease (CVD) are serious public health issues. IR has been proven to have an important impact on diabetes and CVD risk factors [16]. Insulin resistance develops when insulin fails to exert its full effect on target tissues. Because of IR, cardiometabolic disorders such as obesity, dyslipidaemia, endothelial dysfunction, and hypertension occur, all of which are risk factors for atherosclerosis and cardiovascular disease [17, 18].

The TyG index is a useful predictor of insulin resistance [19]. The TyG index has been identified as a valuable diagnostic marker for predicting metabolic syndrome [20]. The TyG index is a valuable measure for assessing glycaemic control in T2DM patients, and it has been found to be capable of identifying those at risk of developing diabetes [3, 21]. Additionally, numerous studies have examined the manner in which the TyG index predicts IR in various groups. A Chinese study found that rural inhabitants with a higher TyG index were more likely to develop T2DM [22]. Alice et al. [23] discovered the TyG index to be useful in predicting the risk of IR and other cardiometabolic risk factors in children and adolescents. TyG has also been found to be independently related to IR as well as unfavourable cardiovascular events in young and middle-aged American populations, with a stronger connection reported in those who were obese [24].

The TyG index has been reported to be positively associated with cardiovascular risk factors [25]. Some studies found that the TyG index was also related to the prevalence of CAD [26, 27]. Subsequently, several investigations have since established a link between the TyG index and atherosclerosis [28, 29]. Lee et al. [30] found an independent connection between the TyG index and coronary artery stenosis in individuals with type 2 diabetes. Huang et al. [31] evaluated the prognostic value of the triglyceride glucose (TyG) index in patients with acute decompensated heart failure. Some studies found a relation between the TyG index and CAD throughout the symptomatic period, regardless of social, clinical, and food consumption factors. Won et al. [32] found that the TyG index is an independent predictor for the progression of CAC, especially in adults without severe baseline CAD. Park et al. [33] reported that the TyG index is an independent marker for predicting subclinical CAD in individuals conventionally considered healthy. Furthermore, those who performed the PURE study found an association between the highest TyG index level in low-income countries and middle-income countries with an elevated risk of cardiovascular mortality, myocardial infarction, stroke, and incident diabetes [34]. As a result, analysing the TyG index is critical, especially regarding the risk of developing CVD.

In a recent study, the TyG index was found to be related to carotid atherosclerosis and arterial stiffness [35, 36]. Alizargar and Bai [37] reported that the TyG index could be used to predict the common carotid artery IMT independent of other risk factors. Carotid plaque is a manifestation of systemic atherosclerosis. Extensive plaque formation and significant lumen constriction result in cerebral ischaemia symptoms, which can lead to stroke in extreme cases. The formation of carotid plaques must be observed to determine the systemic atherosclerotic status. We performed carotid ultrasonography on individuals to investigate the link between the TyG index and carotid lesions using the carotid intima-media thickness and plaque thickness. To the best of our knowledge, there have been few studies on the relationship between the TyG index and the presence of carotid and coronary atherosclerosis among patients with symptomatic CAD.

In this investigation, we included Chinese patients with symptomatic cardiovascular disease who were hospitalized at Tianjin Union Medical Center. We observed a relation between the TyG index and the prevalence of atherosclerosis in patients with symptomatic CAD. After adjusting for sex, age, smoking, BMI, hypertension, diabetes, and the use of antilipemic and antidiabetic agents, the risk of developing coronary lesions and carotid plaques increased across the baseline TyG index. Compared with the lowest TyG index quartile, the highest quartile (quartile 4) was associated with a greater incidence of CHD [OR = 2.55 (95% CI 1.61–4.03)] and carotid atherosclerotic plaque [OR = 2.31 (95% CI 1.27, 4.20)] (*P* < 0.05). In the symptomatic CAD patients, the TyG index showed a significant positive correlation with both coronary lesions and carotid plaques and is of greater value for the identification of both CHD and carotid plaque than the FBG or TG level alone.

Fasting glucose and triglyceride levels are used to compute the TyG index. As a result, these two indices have an impact on the value. Furthermore, diabetes and hyperlipidaemia are separate risk factors for coronary heart disease. To show that the TyG index and atherosclerosis are connected irrespective of glucose and lipid levels, we divided the research population into subgroups based on the existence of diabetes and hyperlipidaemia. The results showed that the TyG index could be used to predict the extent of coronary and carotid artery disease equally well regardless of diabetes and hyperlipidaemia. Separate analysis of the carotid and coronary artery lesions indicated that the TyG index can be used to predict arteriosclerosis from a peripheral and central vascular perspective.

There are several limitations to the present study. First, the sample size may not be sufficiently large. Second, as this was a retrospective observational study, the results could have been affected by memory deficits and inaccurate descriptions of symptoms. Third, other confounding factors, such as exercise habits and job category, were not included. Finally, we cannot adjust for nutritional habits, which can affect blood glucose and triglyceride levels. Therefore, more extensive experiments on this topic are needed in the future.

## Conclusions

Regardless of conventional influencing factors, there was a positive relation between the TyG index and atherosclerosis. According to the results of this study, the TyG index could serve as a good marker for predicting coronary and carotid lesions in patients with symptomatic CAD, regardless of diabetes and hyperlipidaemia. More large-scale prospective investigations are needed to clarify the mechanisms underlying this association.

## Data Availability

The datasets used and/or analysed during the current study are available from the corresponding author on reasonable request.
